# Supplementary material for: Use of Venetoclax in Patients with Relapsed or Refractory Acute Myeloid Leukemia: The PETHEMA Registry Experience
Source: Cancers (Basel). 2022 Mar 29;14(7):1734. doi: 10.3390/cancers14071734 (PMC8997036; doi:10.3390/cancers14071734)
Supplement: Supplementary file 1 [file cancers-14-01734-s001.zip › cancers-1629351-supplementary.pdf]

cancers

MDPI

Table S1. Coagulation, liver enzymes and renal function patients' characteristics prior to venetoclax treatment.

Variable	All patients		Azacitidine + venetoclax		Decitabine + venetoclax		Low-dose cytarabine + venetoclax	
	n (%)	Median (range)	*n* (%)	Median (range)	*n* (%)	Median (range)	*n* (%)	Median (range)
Total	51(100)		30(58.8)		15(29.4)		6 (11.8)	
Platelet count (×10⁹/L)	48	26 (2–188)	29	27 (4–179)	13	26 (7–188)	6	14.5 (2–43)
Platelet < 20 × 10⁹/L	19(39.6)		19(65.5)		8(61.5)		4(33.3)	
PT or APTT times	37		23		10		4	
Normal	26 (70)		17(73.9)		7 (70)		2 (50)	
Prolonged	11 (30)		6(26.1)		3 (30)		2 (50)	
Creatinine (mg/dL)	49	0.82(0.34–1.70)	30	0.74(0.34–1.70)	13	0.91(0.53–1.40)	6	0.85(0.76–1.40)
Uric acid (mg/dL)	46	4.0 (0.2–9.0)	27	4.1 (1.3–9.0)	13	4.0 (0.2–8.3)	6	4.5 (2.8–6.9)
Bilirubin (mg/dL)	45	0.5 (0.1–1.7)	29	0.5 (0.2–1.7)	10	0.6 (0.1–0.9)	6	0.6 (0.2–1.0)
AST (U/L)	51	20 (7–115)	30	21 (7–86)	15	15 (7–109)	6	25 (14–115)
ALT (U/L)	51	21 (9–251)	30	25 (9–86)	15	16 (10–222)	6	15 (9–102)
ALP (U/L)	51	65 (42–317)	30	74 (55–258)	15	43 (42–317)	6	75 (62–157)
LDH (U/L)	51	210 (74–1029)	30	205 (72–1029)	15	207 (129–765)	6	370 (169–765)
Albumin (g/dL)	36	3.4 (2.3–4.8)	21	3.5 (2.3–4.8)	11	3.2 (2.5–4.1)	4	3.6 (3.0–4.1)

Abbreviations: ALP, Alkaline phosphatase; ALT, Alanine aminotransferase; AST, Aspartate aminotransferase; APTT, Activated partial thromboplastin time; LDH, Lactate dehydrogenase; PT, prothrombin time.

Table S2. Univariate analyses of factors influencing response to venetoclax in R/R-AML.

Variable	CR/CRi		ORR (CR/CRi + PR)	
	***n* (%)**	***p***	***n* (%)**	***p***
Total, *n* = 48	6 (12.5)	-	11 (22.9)	
Gender (male), *n* = 31	4 (12.9)	1.000	8 (25.8)	
Age				
<65y, *n* = 17	0 (0)	0.077	1 (5.9)	0.070
≥65y, *n* = 31	6 (19.4)		10 (32.3)	
Secondary AML				
No, *n* = 28	4 (14.3)	1.000	5 (17.9)	0.312
Yes, *n* = 19	2 (10.5)		6 (31.6)	
AML status				
Relapsed, *n* = 27	4 (14.8)	0.683	5 (18.5)	0.498
Refractory, *n* = 21	2 (9.5)		6 (28.6)	
Refractory to any line prior VEN				
No, *n* = 19	4 (21.1)	0.197	4 (21.1)	1.000
Yes, *n* = 29	2 (6.9)		7 (24.1)	
Refractory to prior HMAs				
No, *n* = 31	4 (12.9)	1.000	7 (22.6)	1.000
Yes, *n* = 17	2 (11.8)		4 (23.5)	
ECOG performance status				
0, *n* = 8	3 (37.5)	0.053	4 (50)	0.073
≥1, *n* = 39	3 (7.7)		7 (17.9)	
WBC count (×10⁹/L)				
<10, *n* = 34	4 (11.8)	1.000	7 (20.6)	0.687
≥10, *n* = 11	1 (9.1)		3 (27.3)	
Platelet count (×10⁹/L)				
≥20, *n* = 18	1 (5.6)	0.634	4 (22.2)	1.000
<20, *n* = 27	4 (14.8)		6 (22.2)	
Bone marrow blast count, %				
<50, *n* = 27	4 (14.8)	0.645	6 (22.2)	1.000
≥50, *n* = 14	1 (7.1)		3 (21.4)	
Myelodysplasia related changes AML				
No, *n* = 15	3 (20)	0.360	4 (26.7)	0.720
Yes, *n* = 33	3 (9.1)		7 (21.2)	
Cytogenetics				
Favorable/Intermediate, *n* = 22	4 (18.2)	0.665	8 (36.4)	0.116
Adverse, *n* = 20	2 (10)		3 (15)	
MRC risk stratification				
Favorable/Intermediate, *n* = 19	4 (21.1)	0.398	6 (31.6)	0.473
Adverse, *n* = 21	2 (9.5)		4 (19)	
ELN 2017 risk stratification				
Favorable/Intermediate, *n* = 5	1 (20)	0.561	1 (20)	1.000
Adverse, *n* = 30	4 (13.3)		7 (23.3)	
Somatic mutations				
NPM1 negative, *n* = 33	3 (9.1)	0.036	6 (18.3)	0.123
NPM1 positive, *n* = 6	3 (50)		3 (50)	
FLT3-ITD negative, *n* = 35	5 (14.3)	0.502	7 (20)	0.223
FLT3-ITD positive, *n* = 4	1 (25)		2 (50)	
CEBPA negative, *n* = 20	1 (5)	0.034	1 (5)	0.002

CEBPA monoalelic, *n* = 3	2 (66.7)		3 (100)	
P53 unmutated, *n* = 20	3 (15)	0.606	6 (30)	1.000
P53 mutated, *n* = 8	2 (25)		2 (25)	
IDH1/2 negative, *n* = 19	2 (10.5)	0.287	5 (19)	1.000
IDH1/2 positive, *n* = 7	2 (28.6)		2 (28.6)	
RUNX1 negative, *n* = 20	4 (20)	0.542	6 (30)	1.000
RUNX1 positive, *n* = 6	0 (0)		1 (16.7)	
ASXL1 negative, *n* = 21	3 (14.3)	1.000	5 (23.8)	0.588
ASXL1 positive, *n* = 5	1 (20)		2 (40)	
Prior treatment with HMAs				
No, *n* = 23	4 (17.4)	0.407	7 (30.4)	0.311
Yes, *n* = 25	2 (8.0)		4 (16.0)	
Prior HSCT				
No, 35	4 (11.4)	0.637	9 (25.7)	0.703
Yes, 12	2 (16.7)		2 (16.7)	

Abbreviations: AML, Acute myeloid leukemia; CR, Complete remission; CRi, CR with incomplete blood count recovery; ELN, European LeukemiaNet; ECOG, Eastern Cooperative Oncology Group scale; FLT3-ITD, fms related receptor tyrosine kinase 3 internal tandem duplications; HMAs, Hypomethilating agents; HSCT, hematopoietic stem cell transplant; MRC, Medical Research Council; NMP1, nucleophosmin 1; PR, Partial response; R/R-AML, relapsed/refractory acute myeloid leukemia; WBC, White blood cell.

Table S3. Factors influencing survival in R/R-AML patients treated with venetoclax.

Variable	Median (days)	*p*-Univariate
Total, *n* = 51	104	
Gender		
Male, *n* = 33	78	0.217
Female, *n* = 18	131	
Age		
<65y, *n* = 17	34	0.919
≥65y, *n* = 34	34	
Secondary AML		
No, *n* = 30	104	0.522
Yes, *n* = 20	104	
AML status		
Relapsed, *n* = 29	78	0.262
Refractory, *n* = 22	131	
Refractory to any line prior VEN		
No, *n* = 21	99	0.825
Yes, *n* = 30	128	
Refractory to prior HMAs		
No, *n* = 34	104	0.976
Yes, *n* = 17	120	
ECOG performance status		
0, *n* = 10	NR	0.001
≥1, *n* = 40	75	
WBC count (×10^9/L)		
<10, *n* = 36	120	0.266
≥10, *n* = 12	69	
Platelet count (×10^9/L)		
≥20, *n* = 19	78	0.576
<20, *n* = 29	120	
Bone marrow blast count, %		
<50, *n* = 28	104	0.261
≥50, *n* = 16	78	
Myelodysplasia related changes AML		
No, *n* = 17	99	0.797
Yes, *n* = 34	104	
Cytogenetics		
Favorable/Intermediate, *n* = 24	99	0.616
Adverse, *n* = 20	104	
MRC risk stratification		
Favorable/Intermediate, *n* = 20	78	0.517
Adverse, *n* = 22	104	
ELN 2017 risk stratification		
Favorable/Intermediate, *n* = 5	43	0.458
Adverse, *n* = 31	104	
Somatic mutations		
NPM1 negative, *n* = 35	104	0.729
NPM1 positive, *n* = 6	99	
FLT3-ITD negative, *n* = 36	99	0.083
FLT3-ITD positive, *n* = 5	NR	

CEBPA negative, *n* = 22	69	0.148
CEBPA monoalelic, *n* = 3	131	
P53 unmutated, *n* = 21	99	0.992
P53 mutated, *n* = 8	104	
IDH1/2 negative, *n* = 19	99	0.552
IDH1/2 positive, *n* = 8	NR	
RUNX1 negative, *n* = 21	104	0.408
RUNX1 positive, *n* = 6	66	
ASXL1 negative, *n* = 21	99	0.674
ASXL1 positive, *n* = 5	NR	
Prior treatment with HMAs		
No, *n* = 25	128	0.489
Yes, *n* = 26	78	
Prior HSCT		
No, 38	99	0.510
Yes, 12	128	
Venetoclax combination		
Azacitidine, *n* = 30	120	0.875
Decitabine, *n* = 15	104	
LDAC, *n* = 6	69	
Response to venetoclax		
CR/CRi, *n* = 6	215	0.008
PR, *n* = 5	144	
< PR, *n* = 37	69	
ORR (CR + CRi + PR)		
No, 37	69	0.004
Yes,11	215	

Abbreviations: AML, Acute myeloid leukemia; CR, Complete remission; CRi, CR with incomplete blood count recovery; ELN, European LeukemiaNet; ECOG, Eastern Cooperative Oncology Group scale; FLT3-ITD, fms related receptor tyrosine kinase 3 internal tandem duplications; HMAs, Hypomethilating agents; HSCT, hematopoietic stem cell transplant; LDAC, Low-dose cytarabine; MRC, Medical Research Council; NMP1, nucleophosmin 1;ORR, Overall response rate; PR, Partial response; R/R-AML, relapsed/refractory acute myeloid leukemia; WBC, White blood cell.
